# Analysis of COI and ITS2 regions of DNA obtained from *Paragonimus westermani* eggs in ancient coprolites on Joseon dynasty mummies

**DOI:** 10.1590/0074-02760180595

**Published:** 2019-05-16

**Authors:** Jong Ha Hong, Chang Seok Oh, Min Seo, Dong Hoon Shin

**Affiliations:** 1 Seoul National University College of Medicine, Laboratory of Bioanthropology, Paleopathology and History of Diseases, Seoul, South Korea; 2 Seoul National University College of Medicine, Institute of Forensic Science, Seoul, South Korea; 3 Dankook University College of Medicine, Department of Parasitology, Cheonan, South Korea

**Keywords:** ancient parasite, Paragonimus westermani, ancient DNA

## Abstract

The genetic information of ancient *Paragonimus westermani*, the oriental lung fluke infecting over 20 million people worldwide, has not been thoroughly investigated thus far. We analysed genetic markers (COI and ITS2) of *P. westermani* from coprolite specimens (n = 6) obtained from 15th to 18th century Korean mummies. Our results indicated that all *P. westermani* sequences were generally distinct from the other species of the genus *Paragonimus*. The sequences were clustered into three groups: Group I for East Asia; Group II for South and Southeast Asia; and Group III for India and Sri Lanka. In this study, we found that ancient *P. westermani* sequences in Korea belong to Group I, adding invaluable information to the existing knowledge of *Paragonimus* paleogenetics.

Paragonimiasis occurs in definitive hosts after ingestion of infected intermediate hosts.^1^ In general, *Paragonimus* eggs hatch before entering snails (the first intermediate hosts). When the cercariae penetrate crustaceans such as crayfishes or crabs (the second intermediate hosts), they encyst in gills, muscles, and viscera, developing into metacercariae. After definitive hosts (e.g. humans) eat the freshwater crustaceans raw, the metacercariae excyst in the small intestine, penetrating the intestinal wall. They then traverse the diaphragm, entering the peribronchiolar tissues of the lung where they mature into adults within 8-12 weeks.^2^

The genus *Paragonimus* constitutes a species-rich group from the tropical regions in South and Southeast Asia, Africa, and Central and South America as well as the temperate zone of North America and East Asia.^3^ Almost 50 nominal species and subspecies of *Paragonimus* have been reported so far;^1^ and approximately 16 species have been revealed to cause human diseases. Paragonimiasis mainly affects the lung. The adult worms stimulate the formation of worm capsules which then ulcerate, potentially leading to clinical symptoms such as cough, blood-tinged sputum, and pulmonary pain. Paragonimiasis could also be induced by the migration of young adult worms to the ectopic organs.^2^ ^,^ ^4^ ^,^ ^5^ ^,^ ^6^ ^,^ ^7^ Among *Paragonimus*, the most common species is *P. westermani*, the oriental lung fluke mainly reported in Korea, China, Taiwan, Japan, and the Philippines.^1^ ^,^ ^8^ ^,^ ^9^ Scholars have estimated that over 20 million people are currently infected with *P. westermani* worldwide.^1^ ^,^ ^10^

Recently, researchers have attempted to reveal the genetic characteristics of *P. westermani* through DNA analysis.^1^ ^,^ ^8^ ^,^ ^11^ ^,^ ^12^ ^,^ ^13^ ^,^ ^14^ ^,^ ^15^ Phylogenetic analyses of cytochrome c oxidase subunit I (COI) and internal transcribed spacer 2 (ITS2) DNA regions have revealed that *P. westermani* are clustered into at least two groups (East Asia and South/Southeast Asia), in association with geographically distinct distributions.^1^ ^,^ ^8^ ^,^ ^11^ ^,^ ^12^ ^,^ ^13^ However, although ancient eggs have been detected in archaeological samples, very few parasitological reports regarding paleogenetics of ancient *Paragonimus* spp. have been published so far. The only report was our previous study on ITS2 DNA sequence of ancient *P. westermani* eggs obtained from a 17th century Korean mummy.^7^ In that study, the ancient DNA sequence of *P. westermani* was very similar to that of modern *P. westermani* reported in East Asia, but was genetically distinct from the *P. westermani* sequences of Southeast Asia.^7^

Nevertheless, our previous study was performed with only one sample and a single genetic marker (ITS2). Over the past several years, the genetic information of another *P. westermani* genetic marker (COI) has been continuously accumulated by multiple researchers.^11^ ^,^ ^12^ ^,^ ^13^ ^,^ ^16^ ^,^ ^17^ Our paleoparasitological studies have also microscopically detected ancient *Paragonimus* eggs in many more coprolite specimens (n = 6) from the 15th to 18th century Korean mummies.^18^ ^,^ ^19^ In this regard, we attempted to examine multiple genetic markers (COI and ITS2) in newly collected ancient *P. westermani* eggs in order to obtain more comprehensive information about the evolutionary history of *P. westermani*.

The samples used in this study were acquired from the 15th to 18th century Joseon-era mummies (Cheongdo, Dangjin, Hwasung, YG2-4, YG2-6, and Yongin). The specimens were ancient faeces from the intestines of mummies (Cheongdo and Hwasung) or the organic materials compiled upon the hipbones of half-mummified bodies (Dangjin, YG2-4, YG2-6 and Yongin).

To authenticate our work on ancient DNA (aDNA), we followed the Criteria of Authentication established by Hofreiter et al.^20^ During aDNA analysis, we wore head caps, masks, protection gloves, and gowns. All the tools used in this study were sterilized before use. We also used specialised facilities that were exclusively dedicated to aDNA analysis.^21^ We performed experiments in this facility that were developed in accordance with the Criteria.^20^ The Institutional Review Board (IRB) of Seoul National University Hospital confirmed that our aDNA analyses could be exempt from board review (IRB No. 2017-001). We also followed the Vermillion Accord on Human Remains adopted by the World Archaeological Congress.^22^

For DNA extraction, we followed the method described in our previous report.^23^ In brief, the samples (0.3 g each) were incubated at 56ºC in 1 mL of lysis buffer (pH 8.0; including 1 % SDS; 0.1M DTT; 50 mM of EDTA; 1 mg/mL of proteinase K) for 24 h. After DNA was extracted with an equal volume of phenol/chloroform/isoamyl alcohol (25:24:1), it was then treated with chloroform/isoamyl alcohol (24:1). DNA isolation and purification were performed using a QIAmp PCR purification kit (Qiagen, Hilden, Germany). The purified DNA was eluted in 40 μl of EB elution buffer (Qiagen, Hilden, Germany). Primers for the *P. westermani* COI and ITS2 regions were generated by Integrated DNA Technologies, Inc. (Iowa City, IA, USA). The information of the primers used in our study is summarised in Table I.

**TABLE I:** List of primers used for amplification of *Paragonimus westermani* DNA in this study

Region	Set	Primer	5’ to 3’	Annealing temp. (ºC)	Length (bp)
COI	COI1	COI-1F	GGG CAT CCG GAG GTG TAT GT	54	106
		COI-1R	TTC GGG TAC TAC GGG CTG G		
	COI2	COI-2F	CTG ACC AAC AAC GAT TCC T	54	150
		COI-2R	TCC CGT GAC AGA ACT AAA GA		
	COI3	COI-3F	GTC TGG GTA GTG TTG TGT GG	54	136
		COI-3R	AGC ATG AAC AAC CAA GAG AA		
	COI4	COI-4F	TTA GTT CTG TCA CGG GAG TG	57	114
		COI-4R	GAA TTC ACC ACA AAA CAG GA		
	COI5	COI-5F	TTC TCT TGG TTG TTC ATG CT	54	183
		COI-5R	GAC GTA ATG AAA ATG AGC C		
ITS2	ITS2-1	ITS2-1F	GCG CAG CCA ACT GTG TGA A	57	133
		ITS2-1R	GGC GTC GCG ATA GTT TAT		
	ITS2-2	ITS2-2F	TTA ATG CGA ACT GCA TAC TG	54	169
		ITS2-2R	AAG ACC AGA TTG GGG AGA T		
	ITS2-3	ITS2-3F	GGT CGG CTT ATA AAC TAT CG	54	129
		ITS2-3R	CCC GAG TAT GTT AGG GAA A		
	ITS2-4	ITS2-4F	AAT CTG GTC TTG TGC CTG T	60	165
		ITS2-4R	AAA CCA CAG ATG AAG ACA GG		
	ITS2-5	ITS2-5F	GTG GCT CAG TGA ATG ATT TAT	54	170
		ITS2-5R	CCG CTT AGT GAT ATG CTT A		

DNA quantification was performed using a NanoDrop^TM^ ND-1000 Spectrophotometer (Thermo Fisher Scientific, MA, USA). Extracted DNA (10 μL) was treated at 37ºC with 1 unit of uracil-DNA-glycosylase (New England Biolabs, MA, USA) for 30 min. It (40 ng) was then mixed with the reagent premix containing 10 pmol of each primer and 1X AmpliTaq Gold® 360 Master Mix (Life Technologies, CA, USA). PCR conditions were as follows: pre-denaturation at 95ºC for 10 min, 45 cycles of denaturation at 95ºC for 30 s, annealing at 54-60ºC for 30 s, extension at 72ºC for 30 s, and final extension at 72ºC for 10 min. The polymerase chain reaction (PCR) products were separated on 2.5% agarose gel (Invitrogen, CA, USA), and then stained with ethidium bromide. Negative controls (extraction controls) were also applied to the electrophoresis at the same time. Electrophoresis results were photographed using a Vilber Lourmat ETX-20.M equipped with Biocapt software (Vilber Lourmat, Collégien, France).

The PCR amplicons were isolated using a QIAquick Gel Extraction Kit (Qiagen, Hilden, Germany). The bacteria were transformed using the pGEM-T Easy Vector system (Promega Corporation, Madison, USA). Transformed bacteria were grown on an agar plate containing X-GAL (40 μg/μL), ampicillin (50 μg/mL), and 0.5 mM IPTG for the 14 h. After selected colonies were grown in LB media for 12 h, the purification of cultured bacteria was performed using a QIAprep® Spin Miniprep kit (Qiagen, Hilden, Germany).

Each amplified strand was sequenced using an ABI Prism BigDye Terminator v3.1 Cycle Sequencing Ready Reaction Kit (Applied Biosystems, Waltham, USA) and the 3730xl Automatic Sequencer (Applied Biosystems, Waltham, USA). The obtained DNA sequences were aligned by MEGA7 program.^24^ The consensus sequences were compared to those available in GenBank using NCBI/BLAST tools.^25^ The web browser module and Alignment Explorer of MEGA7 were used to retrieve sequences homologous to those of interest from NCBI GenBank database.

The evolutionary relationship of *P. westermani* and other taxa from NCBI GenBank was inferred from the Phylogeny Reconstruction analysis implemented in MEGA7.^24^ We used the Maximum Likelihood (ML) method. Selected parameters of our method followed the Tamura-Nei model^26^ for COI or the Kimura 2-parameter model^27^ for ITS2; we selected Complete Deletion for Gaps/Missing data treatment, Uniform Rates for Rates among Sites, and Nearest-Neighbor-Interchange (NNI) for the ML Heuristic Method. To estimate the reliability of the tree, we performed a bootstrap test.^28^ The number of bootstrap replicates was 1000.

To select the specimens used for this experiment, we screened all the mummy coprolite samples using PCR with *P. westermani-*specific primers (COI1 and ITS2-1 in Table I). In agarose gel electrophoresis, the amplified products specific for *P. westermani* primer sets COI1 (106 bp) and ITS2-1 (133 bp) were detected in only two samples (Cheongdo and YG2-4) while negative controls (extraction controls) did not exhibit any amplified bands (Fig. 1). Thus, we used Cheongdo and YG2-4 mummy specimens for subsequent experiments.

**Fig. 1:**
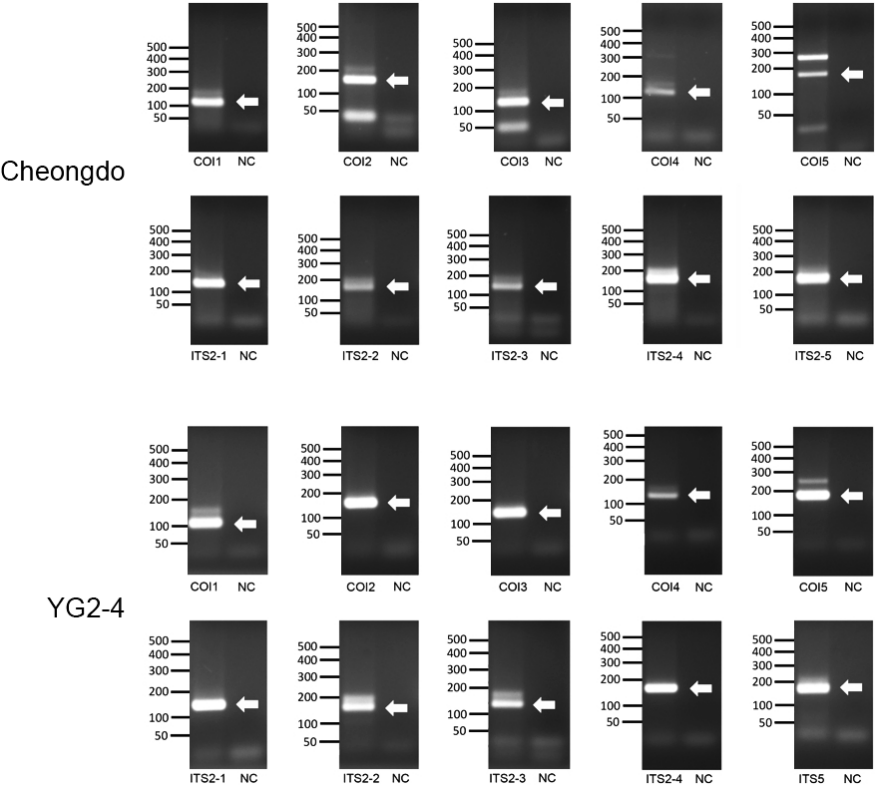
agarose gel electrophoresis for the polymerase chain reaction (PCR) amplicons of *Paragonimus westermani* aDNA from Cheongdo and YG2-4 mummies. Note specific amplicons 1 (106 bp), 2 (150 bp), 3 (136 bp), 4 (114 bp), and 5 (183 bp) of the COI region and 1 (133 bp), 2 (169 bp), 3 (129 bp), 4 (165 bp), and 5 (170 bp) of ITS2 region.

To obtain the consensus sequences of COI and ITS2 regions, we repeated cloning and sequencing for each specific amplicon. From these trials, 9-12 clone sequences were successfully acquired for the COI (390 bp) and ITS2 (417 bp) regions. Next, multiple sequence alignment was performed using Clustal W implemented in MEGA7. We then obtained consensus sequences after alignment of the cloned sequences [Supplementary data (Fig. 1)]. The consensus sequences of the *P. westermani* COI and ITS2 regions from the Cheongdo and YG2-4 mummies were identical [Supplementary data (Fig. 2)].

Using NCBI/BLAST tools, we compared the consensus sequences with those available in GenBank [Supplementary data (Fig. 2)]. The BLAST results are summarised in Table II. Briefly, the current *P. westermani* COI sequences obtained from Joseon mummies exhibited significant similarities to the *P. westermani* COI sequences reported in Korea (AF333280.1; AF540958.1), Japan (U97205.1; U97208.1), China (AY140680.1; U97209.1), Vietnam (FJ434988.1), India (JN656169.1; KM280646.1), the Philippines (U97213.1), Thailand (AB354224.1), and Sri Lanka (AY240940.1). We also found moderate similarities to the GenBank sequences of *P. siamensis* (AB354231.1), *P. paishuihoensis* (AB679289.1), and *P. mexicanus* (KC562301.1) [Supplementary data (Fig. 2A); Table II].

**TABLE II:** BLAST searching results for the coverage and percent identity of each taxon comparing to the consensus sequence of *Paragonimus westermani* COI and ITS2 from Korean mummies. GenBank accession numbers and geographical information are also indicated

DNA region	Species	Coverage	Percent identity	Accession number	Geographical region
COI	*P. westermani*	100%	100%	U97205.1	Japan
		100%	99%	AF333280.1	South Korea
		100%	99%	AF540958.1	South Korea
		100%	99%	U97208.1	Japan
		98%	99%	AY140680.1	China
		100%	98%	U97209.1	China
		99%	96%	FJ434988.1	Vietnam
		100%	93%	JN656169.1	India
		99%	86%	KM280646.1	India
		99%	90%	U97213.1	The Philippines
		99%	89%	AB354224.1	Thailand
		92%	84%	AY240940.1	Sri Lanka
	*P. siamensis*	99%	95%	AB354231.1	Thailand
	*P. paishuihoensis*	99%	84%	AB679289.1	Laos
ITS2	*P. westermani*	100%	100%	KC417492.1	China
		100%	100%	AB713404.1	China
		100%	100%	AB938198.1	India
		100%	99%	AF333277.1	South Korea
		100%	99%	JN656205.1	India
		100%	99%	JN656208.1	India
		100%	99%	U96908.1	Taiwan
		100%	99%	LC144902.1	Vietnam
		100%	99%	AB354217.1	Thailand
		100%	98%	DQ836243.1	India
		100%	98%	JN656199.1	India
		100%	97%	DQ351845.1	India
		100%	97%	AB354214.1	Thailand
		97%	99%	FJ434982.1	Vietnam
		87%	100%	JF500452.1	Ancient South Korea
		87%	100%	U96907.1	Japan
		87%	98%	U96909.1	Malaysia
		87%	97%	AF159604.1	Thailand
		68%	95%	AY240942.1	Sri Lanka
	*P. siamensis*	100%	96%	AB354222.1	Thailand
	*Euparagonimus cenocopiosus*	87%	93%	AF159601.1	China

In the case of the ITS2 region, the current Joseon *P. westermani* sequences exhibit similarities to *P. westermani* ITS2 sequences found in Korea (AF333277.1), Japan (U96907.1), China (KC417492.1; AB713404.1), India (AB938198.1; JN656208.1; DQ836243.1), Taiwan (U96908.1), Vietnam (LC144902.1; FJ434982.1), Malaysia (U96909.1), Thailand (AB354217.1; AF159604.1), and Sri Lanka (AY240942.1). Similar sequences were also found in the sequences of *P. siamensis* (AB354222.1), *Euparagonimus cenocopiosus* (AF159601.1), and *P. skrjabini* (AB703444.1). The *P. westermani* ITS2 sequence obtained from a Korean mummy reported in our previous study (Shin et al.^7^ JF500452.1) also demonstrated 100% identity (coverage 87%) [Supplementary data (Fig. 2B); Table II].

Next, we performed phylogenetic analysis of *P. westermani* COI and ITS2 regions (Fig. 2). Every *P. westermani* taxon in the COI and ITS2 regions was clearly distinct from other *Paragonimus* species (*P. siamensis*, *P. skrjabini*, *P. paishuihoensis* and *P. mexicanus*). *P. westermani* sequences were clustered into several groups. In the case of the COI region, Group I included *P. westermani* sequences from East Asia (Korea, Japan, and China) whereas Group II included those from South and Southeast Asia (India, Philippines, and Thailand) and formed another cluster. We note that some sequences reported from India and Sri Lanka formed a separate cluster of the third separate group (Group III) (Fig. 2A). *P. westermani* COI sequences obtained from Korean mummies in this study evidently belong to the East Asia group. Our result was similar to those of the phylogenetic analyses conducted by Iwagami et al.^13^ ^,^ ^17^ and Devi et al.,^8^ ^,^ ^16^ which yielded that the *P. westermani* COI region was clustered into East, Southeast, and South Asia groups (Fig. 2A).

**Fig. 2:**
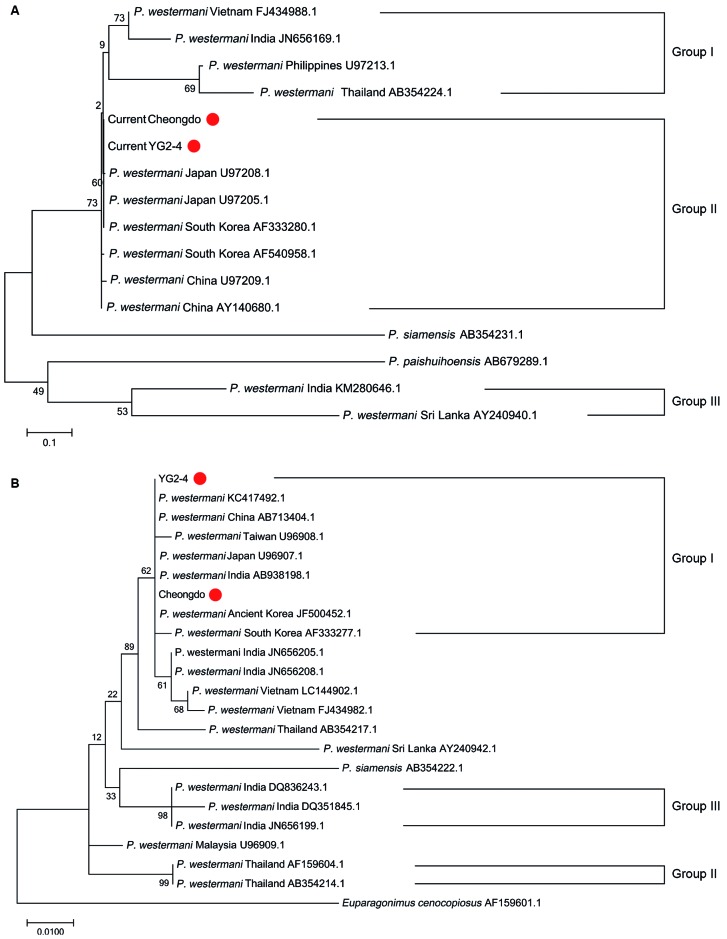
maximum likelihood tree of *Paragonimus* (A) COI and (B) ITS2 DNA region. The percentage of replicate trees in which the associated taxa clustered in the bootstrap test are marked next to the branches. The ancient *P. westermani* sequences revealed in this study are represented by red dots.

Like the COI region, *P. westermani* ITS2 sequences reported from East Asia (Korea, Japan, and China; Group I), Southeast Asia (Thailand; Group II), and South Asia (India; Group III) were also separately clustered in the phylogenetic analysis (Fig. 2B). The *P. westermani* ITS2 sequences from Cheongdo and YG2-4 Korean mummies in our study belonged to the East Asia group as well. Taken together, our phylogenetic tree of the ITS2 region was generally similar to those reported in previous studies conducted by Iwagami et al.^13^ ^,^ ^17^ and Devi et al.^8^ ^,^ ^16^ However, different from the COI region, there was also a unique finding of our examination of the ITS2 region of *P. westermani*. In brief, some *P. westermani* ITS2 sequences from South Asia (India: AB938198.1, JN656208.1, JN656205.1) and Southeast Asia (Vietnam: LC144902.1, FJ434982.1) were clustered with those of the East Asia group in this study (Fig. 2B). Similar findings have rarely been reported except for the research conducted by Doanh et al.^12^

According to the paleoparasitological estimation, the prevalence of *P. westermani* infection in the Joseon society might reach as high as 33.3%.^18^ ^,^ ^19^ Since Joseon people commonly enjoyed raw crayfish or raw crab dishes,^7^ ^,^ ^29^ ^,^ ^30^ it is understandable that they were heavily infected by *P. westermani*. In this study, using coprolite samples obtained from Joseon dynasty mummies, we successfully analysed the COI and ITS2 regions of aDNA from *P. westermani*. As very few studies have examined ancient *P. westermani* DNA so far,^7^ the current study significantly expands the existing gene pool of *P. westermani* paleogenetics. Nevertheless, we admit that aDNA reports of much wider geo-historical scope are still required to improve our knowledge about the exact evolutionary history of *P. westermani*.
