# Supplementary material for: Analysis of COI and ITS2 regions of DNA obtained from Paragonimus westermani eggs in ancient coprolites on Joseon dynasty mummies
Source: Mem Inst Oswaldo Cruz. 2019 May 16;114:e180595. doi: 10.1590/0074-02760180595 (PMC6524962; doi:10.1590/0074-02760180595)
Supplement: Supplementary file 1 [file 1678-8060-mioc-114-e180595-s.pdf]

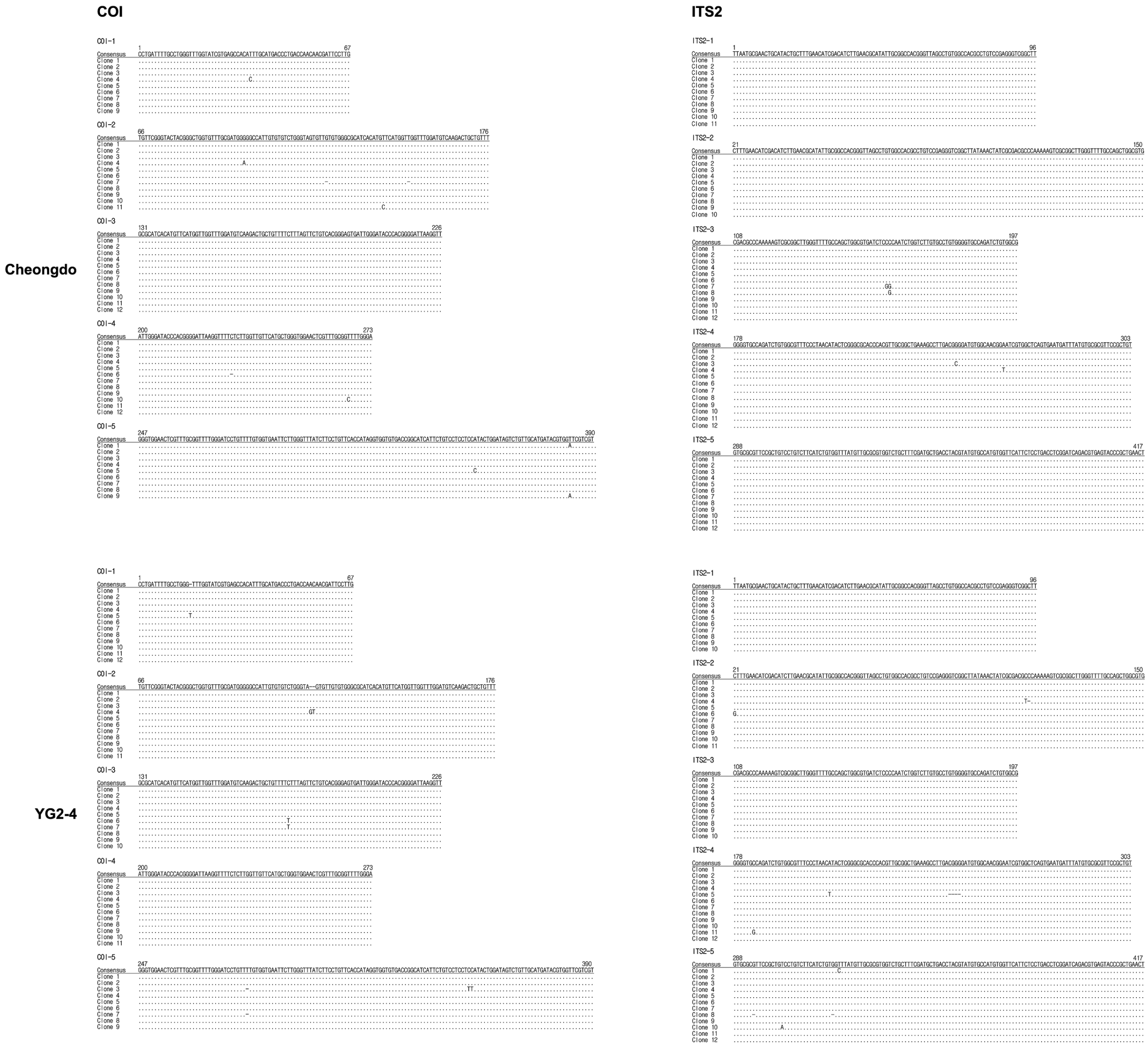

Fig. 1: sequence alignment of cloned polymerase chain reaction (PCR) amplicons for the COI and ITS2 regions of *Paragonimus westermani* aDNA from Cheongdo and YG2-4 mummies.

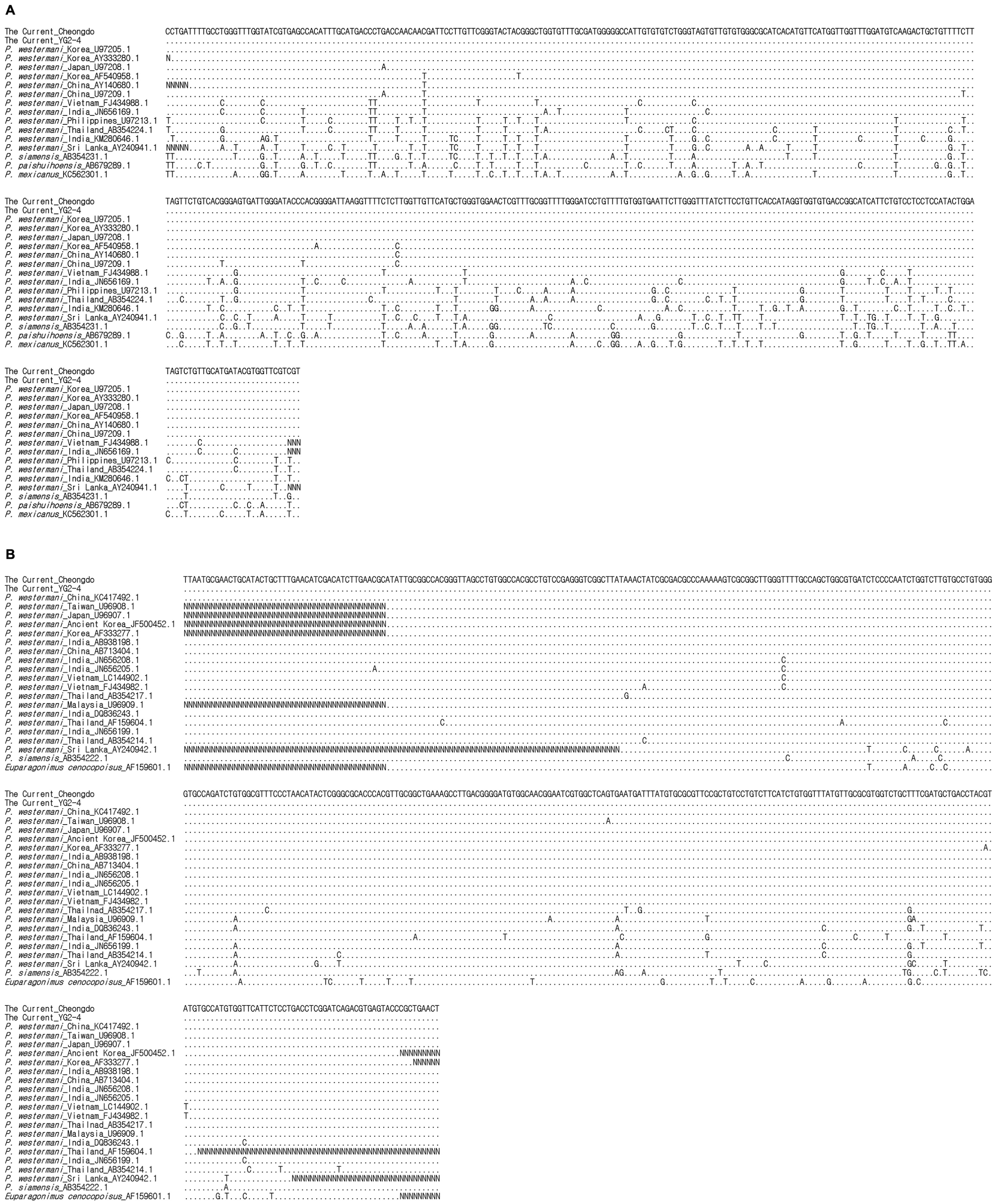

Fig. 2: BLAST comparison of *Paragonimus westermani* consensus sequences from Cheongdo and YG2-4 mummies with the other sequences retrieved at GenBank. (A) and (B) are COI and ITS2 region sequences, respectively.
